# Supplementary material for: Gain engineering and atom lasing in a topological edge state in synthetic dimensions
Source: Nat Commun. 2025 Dec 13;17:421. doi: 10.1038/s41467-025-67106-8 (PMC12795819; doi:10.1038/s41467-025-67106-8)
Supplement: Supplementary file 1 — Supplementary Information [file 41467_2025_67106_MOESM1_ESM.pdf]

Supplementary Information for "Gain engineering and atom lasing in a topological edge state in synthetic dimensions"

Takuto Tsuno,[1,*] Shintaro Taie,[1,*] Yosuke Takasu,[1,†] Kazuya Yamashita,[1,‡] Tomoki Ozawa,[2] and Yoshiro Takahashi[1]

[1]*Department of Physics, Graduate School of Science, Kyoto University, Kyoto 606-8502, Japan*
[2]*Advanced Institute for Materials Research (WPI-AIMR), Tohoku University, Sendai, Miyagi 980-8577, Japan*

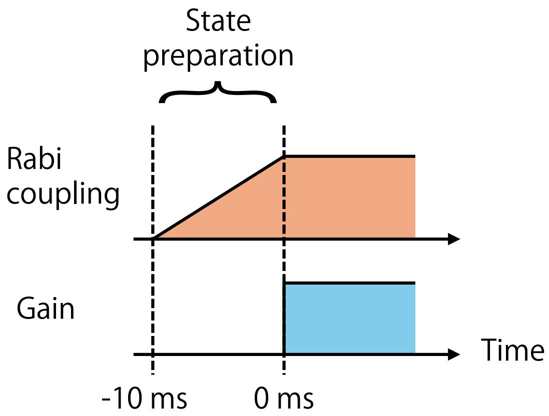

FIG. S1: **Time sequence for numerical simulation.** Since we also want to take into account the adiabaticity of the state preparation, we first prepare the initial state with the thermal atoms at site 5 only. The Rabi couplings are then slowly turned on over 10 ms. Then, the Rabi couplings are kept constant and gain and loss are turned on.

* These two authors contributed equally to this work.
† Electronic address: takasu@scphys.kyoto-u.ac.jp
‡ Present address: Center for Quantum Information and Quantum Biology, The University of Osaka, Toyonaka, Osaka 560-0043, Japan
